# Predictors of COVID-19 Preventive Perceptions and Behaviors Among Millennials: Two Cross-sectional Survey Studies

**DOI:** 10.2196/30612

**Published:** 2021-08-12

**Authors:** Christopher E Beaudoin, Traci Hong

**Affiliations:** 1 College of Communication Boston University Boston, MA United States

**Keywords:** COVID-19, coronavirus, pandemic, preventive perceptions, preventive behaviors, health information seeking, political party identification, COVID-19 testing

## Abstract

**Background:**

COVID-19 preventive perceptions and behaviors, especially among US millennials, are an important means by which the pandemic can be slowed and negative health outcomes can be averted.

**Objective:**

This manuscript aims to advance knowledge on COVID-19 preventive perceptions and behaviors and their main predictors, including digital health information–seeking behavior (HISB), political party identification, and COVID-19 testing status.

**Methods:**

Two cross-sectional online surveys of US millennials were conducted from April 10 to 14, 2020 (N=274) (ie, Study 1), and from April 27 to May 7, 2020 (N=1037) (ie, Study 2). In the regression models, dependent variables included preventive behaviors (eg, wearing a face mask and social distancing) as well as four preventive perceptions: severity (ie, a person’s conception of the seriousness of COVID-19), susceptibility (ie, a person’s conception of the likelihood of being infected with COVID-19), self-efficacy (ie, a person’s perception that he or she can wear a face mask and perform social distancing to prevent COVID-19 infection), and response efficacy (ie, a person’s perception of whether wearing a face mask and social distancing can prevent COVID-19 infection). Key independent variables included digital HISB for self, digital HISB for another person, political party identification, and COVID-19 testing status.

**Results:**

Millennials reported lower levels of perceived susceptibility than the other three preventive perceptions (ie, severity, self-efficacy, and response efficacy), as well as fairly high levels of preventive behaviors. Unlike HISB for another person, digital HISB for self was positively associated with preventive perceptions and behaviors. In Study 1, respondents with higher levels of digital HISB for self had significantly higher perceptions of severity (*β*=.22, *P*<.001), self-efficacy (*β*=.15, *P*=.02), and response efficacy (*β*=.25, *P*<.001) as well as, at nearing significance, higher perceptions of susceptibility (*β*=.11, *P*=.07). In Study 2, respondents with higher levels of digital HISB for self had significantly higher perceptions of severity (*β*=.25, *P*<.001), susceptibility (*β*=.14, *P*<.001), and preventive behaviors (*β*=.24, *P*<.001). Preventive behaviors did not vary significantly according to political party identification, but preventive perceptions did. In Study 1, respondents who identified as being more Republican had significantly lower perceptions of self-efficacy (*β*=−.14, *P*=.02) and response efficacy (*β*=−.13, *P*=.03) and, at nearing significance, lower perceptions of severity (*β*=−.10, *P*=.08) and susceptibility (*β*=−.12, *P*=.06). In Study 2, respondents who identified as being more Republican had significantly lower perceptions of severity (*β*=−.08, *P*=.009). There were mixed effects of COVID-19 testing status on preventive perceptions, with respondents who had tested positive for COVID-19 having significantly higher perceptions of susceptibility in Study 1 (*β*=.17, *P*=.006) and significantly lower perceptions of severity in Study 2 (*β*=−.012, *P*<.001).

**Conclusions:**

As the largest and most digitally savvy generation, US millennials saw COVID-19 as a severe threat, but one that they were less susceptible to. For millennials, digital HISB for self, but not for another person, was critical to the development of preventive perceptions and behaviors.

## Introduction

### Background

COVID-19 is a major global health threat, with 155 million global cases and 3.2 million related deaths as of May 6, 2021, including 32 million cases and 580,012 deaths in the United States [1]. Two pandemic time frames are especially pertinent to this manuscript: April 10 to 14, 2020, and April 27 to May 7, 2020. By April 14, there were 1.8 million confirmed coronavirus cases and 120,548 related deaths globally, including 582,594 confirmed cases and 23,649 deaths in the United States [2]. By May 7, there were 3.72 million confirmed coronavirus cases and 263,489 related deaths, including 1.23 million confirmed cases and 73,431 deaths in the United States.

Before and across these time frames, there were school and workplace closures and stay-at-home orders across the United States, as well as recommendations of preventive measures, such as social distancing and face-mask wearing. In its declaration of COVID-19 as a pandemic in March 2020, the World Health Organization labeled the plethora of coronavirus information as an *infodemic* [3], which stresses the importance of health information. Without effective vaccines prior to 2021, a significant challenge for policy and practical intervention has been encouraging individuals to adopt preventive behaviors (eg, wearing face masks and social distancing) as a means of preventing the virus’ continued spread and the further escalation of negative outcomes. To identify what types of individuals are most likely—and least likely—to adopt COVID-19 preventive behaviors, this manuscript investigates preventive perceptions and behaviors and their predictors, including digital health information–seeking behavior (HISB), political party identification, and COVID-19 testing status.

With implications for theory, policy, and practical intervention, this study focuses on COVID-19 preventive perceptions and behaviors of US millennials, who have been an especially important population for COVID-19 preventive efforts [4], given their high levels of social activity and tendency to have no or mild symptoms [5]. According to Dr Deborah Birx, the White House coronavirus response coordinator, millennials are “the core group that will stop this virus” [6]. Millennials make up the largest generational segment in the United States in terms of both the population and workforce [7,8] and are considered *digital natives* [9], being lifelong users of internet and digital media and the most active and experienced generation in terms of new and emerging technologies [10].

### Predictors of Preventive Perceptions and Behaviors

#### Theoretical Basis

Health behavioral theory provides a framework for understanding psychological processes by which people confront health threats such as COVID-19. The Extended Parallel Process Model (EPPM) entails two predictive processes: danger control and fear control [11]. EPPM postulates that health messaging can influence four types of preventive perceptions (ie, severity, susceptibility, self-efficacy, and response efficacy), with these effect processes determining whether individuals enter danger control or fear control. Individuals who develop sufficient levels of these four perceptions are expected to enter danger control, develop protection motivation, undergo an adaptive response, and adopt preventive behaviors. In EPPM, preventive behavior is an “action to prevent, detect, or control illness conditions” (page 76 in Skinner et al [12]). COVID-19 preventive behaviors include wearing a face mask, staying at home, avoiding public or crowded places, avoiding travel, avoiding contact with high-risk individuals, washing or sanitizing hands, and social distancing [13,14]. Perceived severity entails a person’s conception of a health threat’s seriousness, whereas perceived susceptibility involves a person’s conception of the likelihood of undergoing the threat [11]. Self-efficacy entails individuals’ perceptions that they can perform a suggested response, whereas response efficacy refers to individuals’ perceptions of whether a response will prevent a threat [11].

With a basis in EPPM, this manuscript employs the following COVID-19 outcomes: preventive behaviors (ie, avoiding travel, avoiding gatherings, staying at home, wearing a face mask, washing hands, social distancing, and sheltering in place) and four preventive perceptions (ie, severity, susceptibility, self-efficacy, and response efficacy). The four preventive perceptions align with recent research that has modeled COVID-19 preventive behaviors. In particular, studies have identified fear and threat components, such as severity and susceptibility [15,16], as well as self-efficacy [15-17] and response efficacy [16], as instrumental in predicting preventive behaviors. Research has documented US millennials’ perceived risk of COVID-19 and practice of social distancing. There is evidence that millennials are less likely to social distance than prior generations, but have higher risk perceptions than other generations [18]. Other research has considered the predictors of COVID-19 preventive perceptions and behaviors, focusing on either HISB [19] or political party identification [20]. To advance this area of research, we hypothesize that both HISB and political party identification are influential in the development of preventive perceptions and behaviors related to infectious disease. We also consider the effects of COVID-19 testing status.

#### Health Information–Seeking Behavior

HISB entails how individuals purposively seek out health information from media and other sources [21] and is a means to coping with a health threat and emotions that result from the threat [22]. This manuscript’s measurement of HISB is specific to digital access via computer, smartphone, or other electronic means. With the advance of new media technologies, HISB has increased among US adults, with their reliance on the internet for health information rising from 41% in 2005 to 66% in 2014 [23]. Among US millennials, internet use expanded from 83% in 2005 to 90% in 2010, which was higher than for other generations [10]. Particular to internet-using US millennials, 92% in 2017 and 91% in 2019 conducted a digital search for health or medical information *for self* [24,25].

Across the COVID-19 pandemic, the internet has been a powerful conduit for COVID-19 information. In one study of social media posts in China, it was documented that the public was attentive to such information, especially given that the pandemic had closed off some normal interpersonal communication conduits [26]. During the pandemic, US adults have commonly accessed coronavirus news and other related mediated information. In late March 2020, 92% reported having *fairly or very closely* followed news about the coronavirus pandemic [27], whereas in early April 2020, 87% reported that the internet had been important or essential to them during the pandemic [28]. HISB is imperative given its documented effects on health-related knowledge, preventive behaviors, social support, and emotional health [29-32]. Specific to US millennials, use of the internet for health information neared saturation (ie, 99.3%) prior to the pandemic, which was markedly higher than for members of Generation X and baby boomers [33]. In the context of COVID-19, one study documented that information receptivity was positively associated with COVID-19 preventive behaviors [15]. Another study found that digital COVID-19 information seeking had positive direct effects on preventive behaviors, as well as indirect effects as mediated by perceived worry [19]. Given this basis in the literature, Hypothesis 1 (H1) states the following: Digital HISB is positively associated with COVID-19 preventive perceptions and behaviors.

The surveys used in this manuscript also permit a nuanced examination of HISB. While most research has operationalized HISB in terms of a person’s HISB *for self*, some research has considered a person’s HISB *for another person* [34]. HISB for self entails a person seeking out health information to address a personal health risk or threat, whereas HISB for another person entails a person seeking out health information to address another person’s health risk or threat. This second type of HISB could entail a person seeking out health information for a family member, friend, or other care recipient. This leads to Research Question 1 (RQ1): Does the relationship between digital HISB and COVID-19 preventive perceptions and behaviors vary according to HISB typology—for self versus for another person?

#### Political Party Identification

Political party identification entails individuals’ identification as Democrat, Republican, or Independent, as well as their strength of identification in the first two regards and their leaning toward Democrat or Republican in the third regard [35]. The opposition of Republicans and Democrats to one another, as well as the divide in related sentiments and mutual antipathy, has broadened in recent decades [36]. This growing division has been instrumental in the politicization of the COVID-19 pandemic, which has manifested itself in public health practices and related misinformation [37] and in the polarization of coverage in partisan news media [38]. Misinformation in the media has led to political divides in terms of what is said by opinion leaders and understood by their constituents, including in regard to whether the pandemic actually exists, whether people should adopt preventive behaviors, whether vaccines are safe, and whether people should get them. In this manner, news and other media content have augmented partisan differences in COVID-19 perceptions and practices.

Exemplifying the partisan nature of COVID-19, research in the United States has found that Republican counties have been much less likely to practice social distancing than Democrat counties, with these county-level differences associated with individuals’ viewership of the conservative Fox News channel [20]. Another study documented that, as compared to liberals, conservatives have reported lower levels of fear of COVID-19 [39]. Given this basis, Hypothesis 2 (H2) is as follows: Political party identification is inversely associated with COVID-19 preventive perceptions and behaviors, with respondents who are more Republican exhibiting lower levels than respondents who are more Democrat.

#### COVID-19 Testing Status

An individual’s COVID-19 testing status can be viewed as an indicator of personal relevance [21]. The presence of personal experiences enhances the social significance of related health information and disease processes and can be operationalized in terms of a person’s testing and diagnosis for a disease and experiencing of symptomology [21].

Across the pandemic, the implementation of COVID-19 testing has been important to documenting who has the virus. As of May 6, 2021, there had been 439 million tests conducted in the United States [1]. As a predictor of COVID-19 preventive perceptions and behaviors, it is unclear whether coronavirus testing status would have a positive or negative effect. On one hand, it could be that individuals who tested positive would have a higher perception of the relevance of the coronavirus and, thus, may have elevated levels of COVID-19 preventive perceptions and behaviors. On the other hand, it could be that individuals who tested positive and did not experience negative results would have diminished levels of COVID-19 preventive perceptions and behaviors. Given this uncertainty, Research Question 2 (RQ2) is as follows: What is the relationship between testing positive for COVID-19 and COVID-19 preventive perceptions and behaviors?

## Methods

### Overview

To address the two hypotheses and two research questions, we implemented two empirical studies. For both studies, data were collected via online self-report survey questionnaires. Study 1 examined the effects of digital HISB for self, political party identification, and COVID-19 testing status on four preventive perceptions. Study 2 examined the effects of digital HISB for self, digital HISB for another person, political party identification, and COVID-19 testing status on preventive behaviors and two preventive perceptions. This two-study approach permitted several benefits, including the replication of results specific to the effects of the key antecedents on perceived severity and susceptibility across two separate time frames in April and May 2020. It also permitted the expansion of results to include effects on perceived self-efficacy and response efficacy in Study 1 and to include effects on preventive behaviors in Study 2.

Institutional Review Board approval for both cross-sectional survey studies was attained at the research university of the authors (Study 1: 5550X; Study 2: 5572X). Prior to the online surveys, respondents were provided with an informed consent statement, which included specification of the survey’s purpose, length, and investigators and an indication that participation was voluntary and respondents could stop answering questions at any point across the questionnaire. Qualtrics hosted both surveys and recruited and compensated respondents. Qualtrics recruits samples from traditional research panels. To guarantee the validity, reliability, and integrity of survey data, Qualtrics checks all IP addresses and implements digital fingerprinting technology. Qualtrics and its sample partners implement various procedures to confirm respondent identity and randomly select respondents from survey panels who appear to meet a study’s articulated population parameters. Crafted in a general manner as a means of decreasing potential selection bias, respondent invitations included email, in-app, and SMS notifications. In these studies, panelists who met the sought-after sampling frame were provided with an opportunity to partake in the survey. After the completion of the survey, Qualtrics created a variable in the data set that it recommended for usage that excluded respondents who refused consent, did not complete the survey, completed it in shorter than one-half the median survey completion time, or did not match the sought-after sample quotas.

### Study 1

#### Sample

The first study used data from a cross-sectional online survey of US adults aged 18 years and older. Interviews were conducted from April 10 to 14, 2020 (N=1014). Qualtrics hosted the survey and derived the sample, aiming to match quotas for the US adult population in terms of age, gender, education, household income, and ethnicity. After excluding incompletes and after Qualtrics adjustments to ensure data quality and to match the sought-after sample quotas (n=393), the final sample was comprised of 1014 completed survey interviews. Given this manuscript’s focus on millennials, the subset of respondents aged 25 to 39 years (N=274) was used for all statistical analyses.

#### Measurement

The survey questionnaire’s item wording for key independent and dependent variables is depicted in Textbox 1 [13,14,25,35,36,40-42]. Instructions across the questionnaire referred to the COVID-19 coronavirus and used the term *the coronavirus* in the specific questions. Three survey items for perceived severity (*α*=.87) and three survey items for perceived susceptibility (*α*=.83) were adapted from prior research [40]. Responses were rated on a 5-point Likert scale as follows: strongly disagree, 1; disagree, 2; neither agree nor disagree, 3; agree, 4; and strongly agree, 5. Six survey items for perceived self-efficacy (*α*=.88) and six survey items for perceived response efficacy (*α*=.89) were adapted from prior research [40]. These items were split across two preventive behaviors: wearing a face mask and social distancing. Responses were rated on a 5-point Likert scale as follows: strongly disagree, 1; disagree, 2; neither agree nor disagree, 3; agree, 4; and strongly agree, 5.

Digital HISB for self was measured specific to the novel coronavirus. Using the basic structure of the HISB question from Health Information National Trends Survey (HINTS) 5, Cycle 3 [25], there was one item that entailed seeking of coronavirus information for oneself *in the past month* via *computer, smartphone, or other electronic means*. Responses were rated on a 6-point scale with six possible responses—never, once, several times, once per week, several times per week, and every day—and, with reference to prior research [43], they were then recoded in terms of days per month (0 to 30).

Other key independent variables included political party identification and COVID-19 testing status. For political party identification [35,36], there was a 6-point scale ranging from 1 (strong Democrat) to 6 (strong Republican). In terms of COVID-19 testing status, a dichotomous (ie, yes or no) measure was instituted for whether respondents had tested positive for COVID-19 [41].

Control demographics included age, education, household income, ethnicity, employment, and gender. In terms of the millennial subsample, age responses were from 25 to 39 years. Education was measured at the ordinal level with seven responses ranging from *less than 8 years* to *postgraduate*. Household income was measured at the ordinal level with nine responses ranging from *US $9999 or less* to *US $200,000 or more*. For ethnicity, a dichotomous variable of *White* or *non-White* was created. For employment, a dichotomous variable for full-time employment was created.

**Survey item wording for key independent and dependent variables.**

***Key independent variables***

COVID-19 testing status (responses were 1 [yes] or 0 [no] for the first question; for the second question, responses were 1 [positive] or 0 [negative]) [41]:

- Have you personally been tested for coronavirus, or not?
- Was your coronavirus test positive or negative?

Digital health information–seeking behavior (HISB) for self (responses were rated on a 6-point scale as follows: never, once, several times, once per week, several times per week, and every day) [25]:

- In the past month, how frequently have you used a computer, smartphone, or other electronic means to look for information about coronavirus FOR YOURSELF?

Digital HISB for another person (responses were rated on a 6-point scale as follows: never, once, several times, once per week, several times per week, and every day) [25,42]:

- In the past month, how frequently have you used a computer, smartphone, or other electronic means to look for information about coronavirus FOR ANOTHER PERSON?

Political party identification (for the first introductory question, responses were Republican, Democrat, Independent, no preference, or other party; for the second question, responses were strong Republican or not very strong Republican; for the third question, responses were strong Democrat or not very strong Democrat; and responses for the fourth question were closer to Republican or closer to Democratic) [35,36]:

- Generally speaking, do you usually think of yourself as a Republican, a Democrat, an Independent, or what?
- (If answered Republican to introductory question) Would you call yourself a strong Republican or a not very strong Republican?
- (If answered Democrat to introductory question) Would you call yourself a strong Democrat or a not very strong Democrat?
- (If answered Independent, no preference, or other party to introductory question) Do you think of yourself as closer to the Republican or Democratic party?

***Dependent variables***

Severity (responses were rated on a 5-point Likert scale ranging from 1 [strongly disagree] to 5 [strongly agree]) [40]:

- I believe that getting the coronavirus is severe.
- I believe that getting the coronavirus has severe negative consequences.
- I believe that getting the coronavirus is extremely harmful.

Susceptibility (responses were rated on a 5-point Likert scale ranging from 1 [strongly disagree] to 5 [strongly agree]) [40]:

- I am at risk for getting the coronavirus.
- It is likely that I will get the coronavirus.
- It is possible that I will get the coronavirus.

Self-efficacy (responses were rated on a 5-point Likert scale ranging from 1 [strongly disagree] to 5 [strongly agree]) [40]:

- I have the ability to do social distancing to prevent getting the coronavirus.
- I am able to do social distancing to prevent getting the coronavirus.
- I can easily do social distancing to prevent getting the coronavirus.
- I can easily wear a face mask to prevent getting the coronavirus.
- I have the ability to wear a face mask to prevent getting the coronavirus.
- I am able to wear a face mask to prevent getting the coronavirus.

Response efficacy (responses were rated on a 5-point Likert scale ranging from 1 [strongly disagree] to 5 [strongly agree]) [40]:

- Social distancing is effective to prevent getting the coronavirus.
- If I do social distancing, I am less likely to get the coronavirus.
- Social distancing works to prevent getting the coronavirus.
- Wearing a face mask is effective to prevent getting the coronavirus.
- If I wear a face mask, I am less likely to get the coronavirus.
- Wearing a face mask works to prevent getting the coronavirus.

Preventive behaviors (responses were 1 [yes] or 0 [no]) [13,14]:

- Because of the coronavirus outbreak, have you decided NOT to travel or have you changed your travel plans?
- Because of the coronavirus outbreak, have you stayed at home instead of going to work, school, or other regular activities?
- Because of the coronavirus outbreak, have you bought or worn a protective face mask?
- Because of the coronavirus outbreak, have you frequently washed your hands with soap and water?
- Because of the coronavirus outbreak, have you tried to stay at least six feet way from other people when outside of your household?
- Because of the coronavirus outbreak, have you sheltered in place?

#### Statistical Analysis

Statistical analysis was conducted with Stata 16 (StataCorp LLC). The internal consistency of composite measures was assessed with Cronbach *α*. Descriptive statistics were calculated and reported for each study variable. To test the predictors of preventive perceptions, we used ordinary least squares (OLS) regression. The dependent variables in the models were severity, susceptibility, self-efficacy, and response efficacy. Independent variables were entered in two hierarchical steps: (1) control demographics and (2) digital HISB for self, political party identification, and tested positive for COVID-19. This second block of variables permitted testing of the hypotheses and answering of the research questions. We calculated variance inflation factors (VIFs) for the regression models to gauge for multicollinearity [44]. For the regression models in Study 1 and Study 2, VIF levels were low (ie, <2.00), which indicates no evidence of multicollinearity [44].

### Study 2

#### Sample

The second study used data from a cross-sectional online survey that targeted millennials, in particular US adults aged 25 to 39 years (N=1037). Interviews were conducted from April 27 to May 7, 2020. Qualtrics hosted the survey and derived the sample, aiming to match quotas for the US adult population in terms of gender, education, and ethnicity. After excluding incompletes and after Qualtrics adjustments to ensure data quality and to match the sought-after sample quotas (n=80), the final sample included 1037 completed survey interviews.

#### Measurement

The survey questionnaire’s item wording for key independent and dependent variables is depicted in Textbox 1. Consistent with Study 1, three survey items for perceived severity (*α*=.84) and three survey items for perceived susceptibility (*α*=.76) were adapted from prior research [40] and had the same Likert scale response options as described above. Preventive behaviors were measured with a 7-item additive index (*α*=.79), with dichotomous (ie, yes or no) questions specific to the following practices: avoiding travel, avoiding gatherings, staying at home, wearing face masks, washing hands, social distancing, and sheltering in place [13,14].

Digital HISB was measured in two ways: for self and for another person. Using the basic structure of the question on HISB for self from HINTS 5, Cycle 3 [25], the measurement was expanded to include HISB for another person, with adaption from HINTS 4, Cycle 1 [42]. Like Study 1, the first item specific to *digital HISB for self* entailed seeking of coronavirus information for oneself *in the past month* via *computer, smartphone, or other electronic means*. The second item specific to *digital HISB for another person* entailed seeking of coronavirus information for another person *in the past month* via *computer, smartphone, or other electronic means*. Like Study 1, responses were rated on a 6-point scale and then recoded in terms of days per month (0 to 30) [43].

Political party identification and COVID-19 testing status were measured with the same survey items as in Study 1. Also like Study 1, control demographics included age, education, household income, ethnicity, employment, and gender.

#### Statistical Analysis

Statistical analysis was similar to Study 1, also using Stata 16 (StataCorp LLC). The internal consistency of composite measures was assessed with Cronbach *α*, and descriptive statistics were reported for all measures. To test the predictors of preventive perceptions and behaviors, we used OLS regression. Dependent variables in the models were severity, susceptibility, and preventive behaviors. Independent variables were entered in two hierarchical steps: (1) control demographics and (2) digital HISB for self, digital HISB for another person, political party identification, and tested positive for COVID-19. The second block of variables allowed for testing of the hypotheses and answering of the research questions. As with Study 1, there was no evidence of multicollinearity in the regression models, which had low VIF levels (ie, <2.00) [44].

## Results

### Study 1

Descriptive statistics appear in Table 1. Of the sample, more than 64% were White, and more than 45% were male. The mean age was more than 32 years, the mode for household income was *$50,000 to $74,999*, and the mode for education was *12 years or completed high school*. Finally, more than 51% of the sample reported full-time employment. Among the key predictors, the average for digital HISB for self was 15 days per month. Average levels of the four preventive perceptions were between 3.13 and 4.05 on the 5-point Likert scale.

**Table 1:** Study 1 descriptive statistics.

Variables			Value (N=274)	
**Control independent variables**				
	Age (years), mean (SD)		32.40 (4.29)	
	Male, n (%)		125 (45.6)	
	White, n (%)		177 (64.6)	
	Full-time employment, n (%)		140 (51.1)	
	**Household income (US $), n (%)**			
		0 to 9999	34 (12.4)	
		10,000 to 14,999	12 (4.4)	
		15,000 to 19,999	20 (7.3)	
		20,000 to 34,999	43 (15.7)	
		35,000 to 49,999	35 (12.8)	
		50,000 to 74,999	70 (25.5)	
		75,000 to 99,999	31 (11.3)	
		100,000 to 199,999	26 (9.5)	
		200,000 or more	3 (1.1)	
	**Education, n (%)**			
		Less than 8 years	4 (1.5)	
		8 to 11 years	24 (8.8)	
		12 years or completed high school	120 (43.8)	
		Post–high school training other than college	21 (7.7)	
		Some college	45 (16.4)	
		College degree	42 (15.3)	
		Postgraduate	18 (6.6)	
**Key independent variables**				
	Digital health information–seeking behavior (HISB) for self^a^, mean (SD)		15.35 (12.47)	
	Political party identification^b^, mean (SD)		3.18 (1.68)	
	Tested positive for COVID-19, n (%)		4 (1.5)	
**Dependent variables^c^, mean (SD)**				
	Severity		4.04 (0.98)	
	Susceptibility		3.13 (1.02)	
	Self-efficacy		4.05 (0.92)	
	Response efficacy		3.98 (0.89)	

^a^HISB was rated on a 6-point scale as follows: never, once, several times, once per week, several times per week, and every day. Responses were then recoded in terms of days per month (0 to 30).

^b^Political party identification was rated on a 6-point scale ranging from 1 (strong Democrat) to 6 (strong Republican).

^c^Responses were rated on a 5-point Likert scale ranging from 1 (strongly disagree) to 5 (strongly agree).

Table 2 depicts the hierarchical regression models for this study. The effects of only control variables can be found in Block 1 in Models 1, 3, 5, and 7. At the level of nearing significance (ie, *P*<.10), age was positively associated with severity (see Model 1), White respondents had higher levels of perceived susceptibility (see Model 3), and household income was positively associated with response efficacy (see Model 7). Finally, full-time employed respondents had lower levels of severity, self-efficacy, and response efficacy, with the final relationship nearing significance (see Models 1, 5, and 7).

The regression results in Table 2 also pertain to H1 and H2 as well as to RQ2 (see Block 2 in Models 2, 4, 6, and 8). Supportive of H1, digital HISB for self was positively associated with severity (*β*=.22, *P*<.001), self-efficacy (*β*=.15, *P*=.02), and response efficacy (*β*=.25, *P*<.001) (see Models 2, 6, and 8). Also, there was a near-significant positive association between digital HISB for self and susceptibility (*β*=.11, *P*=.07) (see Model 4). In each case, respondents with higher levels of digital HISB for self had higher perception levels. Supportive of H2, political party identification was negatively associated with self-efficacy (*β*=−.14, *P*=.02) and response efficacy (*β*=−.13, *P*=.03) (see Models 6 and 8). Notably, political party identification also had near-significant inverse associations with severity (*β*=−.10, *P*=.08) and susceptibility (*β*=−.12, *P*=.06) (see Models 2 and 4). In each of the four cases, respondents who identified as being more Republican had lower perception levels. Finally, in terms of RQ2, respondents who tested positive for COVID-19 had higher levels of susceptibility (*β*=.17, *P*=.006) (see Model 4).

**Table 2:** Hierarchical regression predictors of COVID-19 preventive perceptions (N=274).

Independent variable			Dependent variable																																	
			Severity^a^									Susceptibility^b^									Self-efficacy^c^									Response efficacy^d^						
			M^e^1		*P* value		M2		*P* value		M3			*P* value		M4		*P* value		M5			*P* value		M6		*P* value		M7			*P* value		M8		*P* value
**Block 1**																																				
	Male, *β*^f^	–.05		.47		–.04		.56		.02			.76		.02		.72		–.01			.93		.00		.97		–.01			.87		.00		.99	
	White, *β*	.05		.42		.09		.13		.12			.06		.15		.01		.05			.39		.10		.12		.05			.38		.10		.09	
	Household income, *β*	–.01		.91		–.03		.67		.05			.48		.05		.55		.05			.51		.04		.58		.14			.07		.12		.13	
	Education, *β*	.07		.32		.04		.62		.08			.29		.05		.49		.07			.33		.04		.60		–.04			.56		–.09		.23	
	Age, *β*	.11		.07		.09		.13		.02			.70		–.01		.86		.07			.24		.05		.46		.06			.34		.03		.58	
	Full-time employment, *β*	–.18		.10		–.15		.02		.07			.34		.06		.38		–.18			.01		–.17		.01		–.13			.06		–.11		.12	
	*R^2^*	0.05		N/A^g^		N/A		N/A		0.04			N/A		N/A		N/A		0.04			N/A		N/A		N/A		0.03			N/A		N/A		N/A	
	*F*	2.48		<.001		N/A		N/A		1.76			.11		N/A		N/A		1.86			.09		N/A		N/A		1.52			.17		N/A		N/A	
**Block 2**																																				
	Digital health information–seeking behavior for self, *β*	N/A		N/A		.22		<.001		N/A			N/A		.11		.07		N/A			N/A		.15		.02		N/A			N/A		.25		<.001	
	Political party identification, *β*	N/A		N/A		–.10		.08		N/A			N/A		–.12		.06		N/A			N/A		–.14		.02		N/A			N/A		–.13		.03	
	Tested positive for COVID-19, *β*	N/A		N/A		.05		.42		N/A			N/A		.17		.006		N/A			N/A		.09		.15		N/A			N/A		.07		.23	
	*R^2^*	N/A		N/A		0.11		N/A		N/A			N/A		0.09		N/A		N/A			N/A		0.09		N/A		N/A			N/A		0.11		N/A	
	∆*R*^2^	N/A		N/A		0.06		N/A		N/A			N/A		0.05		N/A		N/A			N/A		4.58		N/A		N/A			N/A		0.08		N/A	
	∆*F*	N/A		N/A		5.91		<.001		N/A			N/A		5.28		<.001		N/A			N/A		4.19		.01		N/A			N/A		8.12		<.001	

^a^With severity as the dependent variable, the two hierarchical models are Models 1 and 2.

^b^With susceptibility as the dependent variable, the two hierarchical models are Models 3 and 4.

^c^With self-efficacy as the dependent variable, the two hierarchical models are Models 5 and 6.

^d^With response efficacy as the dependent variable, the two hierarchical models are Models 7 and 8.

^e^M: Model.

^f^*β* values represent the standardized coefficients.

^g^N/A: not applicable for the first model in each dependent variable’s pair of hierarchical regression models, or not applicable for reported *R*^2^, *∆R*^2^, *F,* or *∆F.*

### Study 2

Descriptive statistics appear in Table 3. Of the sample, more than 63% were White and more than 46% were male. The mean age was almost 32 years, the mode for household income was *US $20,000 to US $34,999*, and the mode for education was *12 years or completed high school*. Finally, 52.3% of the sample reported full-time employment. Among the key predictors, the mean for digital HISB for self was more than 13 days per month, whereas the mean for digital HISB for another person was more than 8 days per month. Average levels of severity and self-efficacy were between 3 and 4 on the 5-point Likert scale, and respondents reported having performed an average of almost six of the seven preventive behaviors, with frequently washing hands ranking the highest.

Table 4 depicts the hierarchical regression models for this study. The effects of only control variables can be found in Block 1 in Models 1, 3, and 5. Household income had significant positive associations with susceptibility and preventive behaviors (see Models 3 and 5). At the level of nearing significance, education and age had positive associations with severity (see Model 1). In addition, education was positively associated with preventive behaviors, and, nearing significance, employment was inversely associated with preventive behaviors (see Model 5).

The regression results in Table 4 also pertain to H1 and H2 as well as to RQ1 and RQ2 (see Block 2 in Models 2, 4, and 6). The results for digital HISB for self and digital HISB for another person relate to H1. Support is limited to digital HISB for self, which was positively associated with severity (*β*=.25, *P*<.001), susceptibility (*β*=.14, *P*<.001), and preventive behaviors (*β*=.24, *P*<.001) (see Models 2, 4, and 6). The effects of digital HISB for another person were nonsignificant in each of these models. Thus, in terms of RQ1, digital HISB for self had significant positive associations with each outcome, while digital HISB for another person did not. Supportive of H2, political party identification had a negative association with severity (*β*=−.08, *P*=.009) (see Model 2). Thus, respondents who identified as being more Republican had lower perceptions of severity. Political party identification did not have significant associations with perceived susceptibility or preventive behaviors. In terms of RQ2, respondents who had tested positive for COVID-19 reported lower levels of perceived severity (*β*=−.012, *P*<.001) (see Model 2).

**Table 3:** Study 2 descriptive statistics.

Variables					Value (N=1037)
**Control independent variables**					
	Age (years), mean (SD)			31.65 (4.18)	
	Male, n (%)			483 (46.58)	
	White, n (%)			657 (63.36)	
	Full-time employment, n (%)			542 (52.27)	
	**Household income (US $), n (%)**				
		0 to 9999	142 (13.69)		
		10,000 to 14,999	59 (5.69)		
		15,000 to 19,999	65 (6.27)		
		20,000 to 34,999	191 (18.42)		
		35,000 to 49,999	171 (16.49)		
		50,000 to 74,999	171 (16.49)		
		75,000 to 99,999	118 (11.38)		
		100,000 to 199,999	86 (8.29)		
		200,000 or more	34 (3.28)		
	**Education, n (%)**				
		Less than 8 years	16 (1.54)		
		8 to 11 years	52 (6.56)		
		12 years or completed high school	358 (34.52)		
		Post–high school training other than college	98 (9.45)		
		Some college	198 (19.09)		
		College degree	246 (23.72)		
		Postgraduate	69 (6.65)		
**Key independent variables**					
	Digital health information–seeking behavior (HISB) for self^a^, mean (SD)			13.14 (12.28)	
	HISB for another person^a^, mean (SD)			8.14 (10.33)	
	Political party identification^b^, mean (SD)			3.23 (1.68)	
	Tested positive for COVID-19, n (%)			58 (5.59)	
**Dependent variables**					
	Severity^c^, mean (SD)			3.84 (1.07)	
	Susceptibility^c^, mean (SD)			3.09 (1.03)	
	**Preventive behaviors**				
		7-item index^d^, mean (SD)	5.86 (1.70)		
		Avoided travel, n (%)	831 (80.14)		
		Canceled large gatherings, n (%)	857 (82.64)		
		Stayed at home, n (%)	830 (80.04)		
		Wore a protective face mask, n (%)	841 (81.10)		
		Frequently washed hands, n (%)	941 (90.74)		
		Stayed six feet away from others, n (%)	911 (87.85)		
		Sheltered in place, n (%)	850 (81.97)		

^a^HISB was rated on a 6-point scale as follows: never, once, several times, once per week, several times per week, and every day. Responses were then recoded in terms of days per month (0 to 30).

^b^Political party identification was rated on a 6-point scale ranging from 1 (strong Democrat) to 6 (strong Republican).

^c^Responses were rated on a 5-point Likert scale ranging from 1 (strongly disagree) to 5 (strongly agree).

^d^The 7-item index is an additive composite variable of the seven individual preventive behaviors.

**Table 4:** Hierarchical regression predictors of COVID-19 preventive perceptions and behaviors (N=1037).

Independent variable			Dependent variable																								
			Severity^a^									Susceptibility^b^									Preventive behaviors^c^						
			M^d^1		*P* value		M2		*P* value		M3			*P* value		M4		*P* value		M5			*P* value		M6		*P* value
**Block 1**																											
	Male, *β*^e^	–.05		.15		–.05		.12		.00			.89		–.01		.86		–.02			.51		–.03		.36	
	White, *β*	.04		.22		.05		.10		.04			.23		.05		.11		–.03			.34		–.02		.46	
	Household income, *β*	.05		.15		.05		.11		.09			.01		.09		.01		.08			.02		.09		.01	
	Education, *β*	.06		.06		.01		.68		.04			.32		.00		.90		.17			<.001		.14		<.001	
	Age, *β*	.06		.06		.04		.22		.01			.75		.01		.81		.01			.63		.00		.88	
	Full-time employment, *β*	–.05		.17		–.04		.22		–.01			.78		–.02		.56		–.06			.08		–.06		.05	
	*R^2^*	0.02		N/A^f^		N/A		N/A		0.01			N/A		N/A		N/A		0.05			N/A		N/A		N/A	
	*F*	2.68		.01		N/A		N/A		2.06			.06		N/A		N/A		8.59			<.001		N/A		N/A	
**Block 2**																											
	Digital health information–seeking behavior (HISB) for self, *β*	N/A		N/A		.25		<.001		N/A			N/A		.14		<.001		N/A			N/A		.24		<.001	
	Digital HISB for another person, *β*	N/A		N/A		.04		.24		N/A			N/A		.06		.10		N/A			N/A		.00		.95	
	Political party identification, *β*	N/A		N/A		–.08		.009		N/A			N/A		–.06		.08		N/A			N/A		–.03		.28	
	Tested positive for COVID-19, *β*	N/A		N/A		–.12		<.001		N/A			N/A		.06		.06		N/A			N/A		.02		.57	
	*R^2^*	N/A		N/A		0.12		N/A		N/A			N/A		0.05		N/A		N/A			N/A		0.11		N/A	
	∆*R*^2^	N/A		N/A		0.10		N/A		N/A			N/A		0.04		N/A		N/A			N/A		0.06		N/A	
	∆*F*	N/A		N/A		29.45		<.001		N/A			N/A		1.19		<.001		N/A			N/A		17.18		<.001	

^a^With severity as the dependent variable, the two hierarchical models are Models 1 and 2.

^b^With susceptibility as the dependent variable, the two hierarchical models are Models 3 and 4.

^c^With preventive behaviors as the dependent variable, the two hierarchical models are Models 5 and 6.

^d^M: Model.

^e^*β* values represent the standardized coefficients.

^f^N/A: not applicable for the first model in each dependent variable’s pair of hierarchical regression models, or not applicable for reported *R*^2^, *∆R*^2^, *F,* or *∆F.*

## Discussion

### Principal Findings

Our analyses depict US millennials’ levels of COVID-19 preventive perceptions and behaviors. Danger control, the beneficial process entailing protection motivation and behavior change, requires heightened levels of preventive perceptions: severity, susceptibility, self-efficacy, and response efficacy [11]. Of these four perceptions, levels of susceptibility were lowest in both studies, suggesting a gap in millennials’ understanding of their potential vulnerability in contracting COVID-19. It is somewhat surprising that respondents had what seem to be low perceptions of the likelihood that they could be infected with COVID-19 given that, across this study’s two time frames (ie, April 10 to 14, 2020, and April 27 to May 7, 2020), the virus was widespread and expected to spread further. Thus, whereas levels of perceived severity, self-efficacy, and response efficacy were near 4.00 on the respective 5-point scales, perceptions of susceptibility lagged behind, posing a gap that could be targeted with preventive messaging. Study 2 also provided a picture of whether millennials had performed COVID-19 preventive behaviors. In particular, more than 80% of millennials reported having performed the seven separate preventive behaviors.

In addition, our analyses identified different types of US millennials who were most likely—as well as least likely—to adopt preventive perceptions and behaviors in the context of the COVID-19 pandemic; in a nutshell, health information seeking matters, at least when it comes to self. Across the two empirical studies, digital HISB for self was positively associated with preventive behaviors and each preventive perception; the association was nearing significance in terms of one of the preventive perceptions (ie, susceptibility) in Study 1, which implemented the smaller of the two survey samples. The positive effects of digital HISB are generally consistent with prior research specific to the COVID-19 pandemic [15,19] and other health contexts [29-32]. For example, research has documented that individuals with higher information receptivity [15] and COVID-19 information seeking [19] are more likely to practice COVID-19 preventive behaviors. The results of our two studies underscore the importance of digital health information in the contemporary media world, where mediated health information is widespread and the technical affordances of digital and social media allow users to search out health information in a manner that is purposive or incidental, unbounded by constraints of location and privacy, and diverse in information content and information sources. These findings are also instructive for practical interventions, suggesting the importance of disseminating credible and purposive preventive information across the pandemic. While there is evidence in the literature of misinformation and its negative influence on COVID-19 knowledge and preventive behaviors [45], the overall effect, as documented in this manuscript, is a favorable one in terms of digital HISB for self. Notably, digital HISB for another person was not a significant predictor of any of the outcomes, which suggests that millennials’ danger control processes are a function of their drive to protect themselves, not other people [11]. That the related processes of millennials are driven by digital HISB for self, but not digital HISB for another person, may relate to research that has documented millennials as being more self-centered than previous generations [46].

Political party identification had negative coefficients in each regression model for preventive perceptions, achieving significance in three models and nearing significance in the other three. In each case, respondents who identified more as being Republican had lower perception levels, which underscores how preventive perceptions are a function of political party identification. The related findings on perceived severity and susceptibility are generally in line with prior research that documented that, as compared to liberals, conservatives have lower levels of fear of COVID-19 [39], with fear considered to be an outcome of elevated perceptions of severity and susceptibility [11]. However, arguably, what matters more is that preventive behaviors did not vary significantly by political party identification. This result differs from one prior county-level study that demonstrated that people in Republican counties were much less likely to practice social distancing than people in Democrat counties [20]. That preventive perceptions vary significantly by political party identification is indicative of the contemporary political divide in the United States and the polarization of news coverage in the partisan media [20,36,38]. While altering the predominant slant of news coverage in Fox News and related conservative media may be impossible, public health interventions that disseminate preventive information via other sources and help build media literacy to depoliticize health topics such as COVID-19 could help narrow the perceptual gap between Republicans and Democrats. Interestingly, though preventive perceptions vary by political party identification, preventive behaviors do not. Nevertheless, given the vast literature that indicates that perceptions drive behavior change, we believe that media can still play an important role in educating the public on politicized health topics such as COVID-19.

The results were less consistent when it came to the indicator of COVID-19 testing status, which was a measure of personal relevance [21]. Respondents who tested positive for COVID-19 had significantly higher perceptions of susceptibility in Study 1 and lower perceptions of severity in Study 2. The first result, which occurred very early in the pandemic when testing was not widely available, makes sense in that respondents who had tested positive would think they were more susceptible to COVID-19. In terms of the significant result in Study 2, it could be that respondents who tested positive for COVID-19 considered this infectious disease to be less severe because they did not experience major negative effects.

### Limitations

Five limitations deserve acknowledgement. First, given this study’s reliance on two cross-sectional data sets, no inferences of causation can be made. Second, self-report survey data have some measurement limitations, including social desirability concerns. Third, while the sample size of Study 2 (N=1037) is sufficient, the smaller sample size of Study 1 (N=274) may pose concerns in terms of elevated sampling error and attenuated statistical power. There is evidence of this in the regression results, where standardized coefficients of a certain size were deemed significant at *P* values of less than .05 in Table 4, but not in Table 2. For this reason, we referred to results that were nearing significance (ie, *P*<.10) in the reporting of results for Study 1. Fourth, dichotomous (ie, yes or no) questions, which have been used in prior research [13,14,41], provide a general picture of behavioral compliance, but not one of behavioral frequency. By using frequency measurement with a continuous scale for each preventive behavior, future research could depict how often millennials perform recommended COVID-19 preventive behaviors. Fifth, given changes across the pandemic in terms of COVID-19 risks and behavioral recommendations, we caution researchers in generalizing these results to other time frames across the pandemic, as well as to populations other than millennials. That said, this manuscript’s use of two data sets helps mitigate some such concerns related to time frame.

### Conclusions

In conclusion, this manuscript depicts US millennials’ levels of COVID-19 preventive perceptions and behaviors, as well as their predictive factors. Understanding these levels and predictive factors has implications for theory, policy, and practical intervention. The analyses in both empirical studies highlight the importance of health information seeking in the face of a global pandemic, as well as the politicization of the COVID-19 pandemic. Future research should continue to investigate related processes in terms of COVID-19 and other pandemics. It could advance this manuscript’s results by using panel survey data to derive inferences of causation and making assessments across later stages of the COVID-19 pandemic.

## Abbreviations

EPPM: Extended Parallel Process Model
H1: Hypothesis 1
H2: Hypothesis 2
HINTS: Health Information National Trends Survey
HISB: health information–seeking behavior
OLS: ordinary least squares
RQ1: Research Question 1
RQ2: Research Question 2
VIF: variance inflation factor

## References

[1] Coronavirus Resource Center (2021). COVID-19 Dashboard.

[2] (2020). Coronavirus pandemic (COVID-19). Our World in Data.

[3] The Department of Global Communications (2020). UN tackles ‘infodemic’ of misinformation and cybercrime in COVID-19 crisis. United Nations.

[4] Wagner M, Macaya M, Yeung J, Renton A, Rahim Z, Isaac L (2020). CDC director urges millennials to follow Covid-19 guidelines. CNN.

[5] Stone W (2020). Younger adults are increasingly testing positive for the coronavirus. NPR.

[6] Lahut J (2020). Trump coronavirus task force coordinator says millennials 'are the core group that will stop this virus'. Business Insider.

[7] Fry R (2020). Millennials overtake Baby Boomers as America's largest generation. Pew Research Center.

[8] Fry R (2018). Millennials are the largest generation in the US labor force. Pew Research Center.

[9] Prensky M (2001). Digital natives, digital immigrants Part 1. On the Horizon.

[10] Kohut A, Taylor P, Keeter S, Parker K, Morin R, Cohn D, Lopez MH, Smith G, Fry R, Wang W, Christian LM, Pond A, Clement S (2010). Millennials: Confident. Connected. Open to Change.

[11] Witte K (1992). Putting the fear back into fear appeals: The extended parallel process model. Commun Monogr.

[12] Skinner C, Tiro J, Champion V, Glanz K, Rimer BK, Viswanath K (2015). The Health Belief Model. Health Behaivor: Theory, Research, and Practice. 5th edition.

[13] Camacho-Rivera M, Islam JY, Vidot DC (2020). Associations between chronic health conditions and COVID-19 preventive behaviors among a nationally representative sample of US adults: An analysis of the COVID Impact Survey. Health Equity.

[14] Breakwell GM, Fino E, Jaspal R (2021). The COVID-19 Preventive Behaviors Index: Development and validation in two samples from the United Kingdom. Eval Health Prof.

[15] Roberts JA, David ME (2021). Improving predictions of COVID-19 preventive behavior: Development of a sequential mediation model. J Med Internet Res.

[16] Rad RE, Mohseni S, Takhti HK, Azad MH, Shahabi N, Aghamolaei T, Norozian F (2021). Application of the protection motivation theory for predicting COVID-19 preventive behaviors in Hormozgan, Iran: A cross-sectional study. BMC Public Health.

[17] Chan DKC, Zhang C, Weman-Josefsson K (2021). Why people failed to adhere to COVID-19 preventive behaviors? Perspectives from an integrated behavior change model. Infect Control Hosp Epidemiol.

[18] Masters NB, Shih SF, Bukoff A, Akel KB, Kobayashi LC, Miller AL, Harapan H, Lu Y, Wagner AL (2020). Social distancing in response to the novel coronavirus (COVID-19) in the United States. PLoS One.

[19] Liu PL (2020). COVID-19 information seeking on digital media and preventive behaviors: The mediation role of worry. Cyberpsychol Behav Soc Netw.

[20] Gollwitzer A, Martel C, Brady WJ, Pärnamets P, Freedman IG, Knowles ED, Van Bavel JJ (2020). Partisan differences in physical distancing are linked to health outcomes during the COVID-19 pandemic. Nat Hum Behav.

[21] Johnson JD, Meischke H (1993). A comprehensive model of cancer-related information seeking applied to magazines. Hum Commun Res.

[22] Lambert SD, Loiselle CG (2007). Health information seeking behavior. Qual Health Res.

[23] Yoon J, Huang H, Kim S (2017). Trends in health information-seeking behaviour in the US foreign-born population based on the Health Information National Trends Survey, 2005 - 2014. Inf Res.

[24] National Cancer Institute (2017). Health Information National Trends Survey (HINTS) 5, Cycle 1.

[25] National Cancer Institute (2019). Health Information National Trends Survey (HINTS) 5, Cycle 3.

[26] Zhao X, Fan J, Basnyat I, Hu B (2020). Online health information seeking using "#COVID-19 Patient Seeking Help" on Weibo in Wuhan, China: Descriptive study. J Med Internet Res.

[27] Jurkowitz M, Mitchell A (2020). Older Americans continue to follow COVID-19 news more closely than younger adults. Pew Research Center.

[28] Vogels EA, Perrin A, Rainie L, Anderson M (2020). 53% of Americans say the internet has been essential during the COVID-19 outbreak. Pew Research Center.

[29] Beaudoin CE, Hong T (2011). Health information seeking, diet and physical activity: An empirical assessment by medium and critical demographics. Int J Med Inform.

[30] Shim M, Kelly B, Hornik R (2006). Cancer information scanning and seeking behavior is associated with knowledge, lifestyle choices, and screening. J Health Commun.

[31] Beaudoin CE, Tao CC (2008). Modeling the impact of online cancer resources on the supporters of cancer patients. New Media Soc.

[32] Jamal A, Khan SA, AlHumud A, Al-Duhyyim A, Alrashed M, Bin SF, Alteraif A, Almuziri A, Househ M, Qureshi R (2015). Association of online health information-seeking behavior and self-care activities among type 2 diabetic patients in Saudi Arabia. J Med Internet Res.

[33] Paige SR, Miller MD, Krieger JL, Stellefson M, Cheong J (2018). Electronic health literacy across the lifespan: Measurement invariance study. J Med Internet Res.

[34] Oh YS (2015). Predictors of self and surrogate online health information seeking in family caregivers to cancer survivors. Soc Work Health Care.

[35] (2021). Party identification. Inter-university Consortium for Political and Social Research (ICPSR).

[36] (2014). Political polarization in the American public. Pew Research Center.

[37] Chen E, Chang H, Rao A, Lerman K, Cowan G, Ferrara E (2021). COVID-19 misinformation and the 2020 US presidential election. HKS Misinf Rev.

[38] Hart PS, Chinn S, Soroka S (2020). Politicization and polarization in COVID-19 news coverage. Sci Commun.

[39] Winter T, Riordan BC, Pakpour AH, Griffiths MD, Mason A, Poulgrain JW, Scarf D (2020). Evaluation of the English version of the Fear of COVID-19 Scale and its relationship with behavior change and political beliefs. Int J Ment Health Addict.

[40] Witte K, Meyer G, Martell D (2001). Effective Health Risk Messages: A Step-By-Step Guide.

[41] Kaiser Family Foundation (2020). KFF Coronavirus Poll – March 2020.

[42] National Cancer Institute (2012). Health Information National Trends Survey (HINTS) 4, Cycle 1.

[43] Hampton KN (2019). Social media and change in psychological distress over time: The role of social causation. J Comput Mediat Commun.

[44] Allison P (2012). When can you safely ignore multicollinearity?. Statistical Horizons.

[45] Lee JJ, Kang K, Wang MP, Zhao SZ, Wong JYH, O'Connor S, Yang SC, Shin S (2020). Associations between COVID-19 misinformation exposure and belief with COVID-19 knowledge and preventive behaviors: Cross-sectional online study. J Med Internet Res.

[46] Twenge JM, Campbell SM (2008). Generational differences in psychological traits and their impact on the workplace. J Manage Psychol.

